# Evaluation of depth perception and association of severity in Glaucoma patients and suspects

**DOI:** 10.1186/s12886-021-02198-6

**Published:** 2021-12-15

**Authors:** Pragati Gautam Adhikari, Madhu Thapa, Manisha Dahal

**Affiliations:** Department of ophthalmology, Maharajgunj Medical Campus, Institute of Medicine, Tribhuvan University, Maharajgunj, 44600 Nepal

**Keywords:** POAG, stereoacuity, titmus, frisby

## Abstract

**Background:**

To evaluate depth perception in Primary open angle glaucoma (POAG), glaucoma suspects compared to controls and to determine the association between depth perception and severity of glaucoma.

**Methods:**

This was a hospital based, comparative, cross-sectional study. The ethical clearance was taken from institutional review committee of Institute of Medicine [Reference no.399 (6–11) E^2^ 077-078]. Agematched, equal number of participants in each group (*N*=20) were evaluated with both Titmus and Frisby stereoacuity tests to measure depth perception as stereopsis threshold in seconds of arc. The participants were selected using the purposive sampling technique.

**Results:**

There was no differences in age, sex, or best corrected visual acuity, intraocular pressure, central corneal thickness (CCT), found among the three groups (POAG, Glaucoma Suspects and Control) respectively. However, there was significant difference in cup disc ratio (CDR) between the groups. Equal number of male and female were there in each group, while in POAG group male to female ratio was 3:2. The mean stereoacuity threshold in control group was 53.5±23.23 seconds of arc with Titmus test and 38.75±18.83 seconds of arc with Frisby stereoacuity test. The difference in threshold was significant between control and glaucoma suspect with Titmus (t=1.991, *p*=0.05) and with Frisby (t=2.114, *p*=0.04). The difference was also significant in POAG group by Titmus (t=3.135, *p*=0.0033) and by Frisby (t=3.014, *p*=0.004). More so, with increasing severity of glaucoma, the mean threshold of stereopsis increased as seen with both Titmus and Frisby Tests (ANOVA, *p* < 0.001)

**Conclusion:**

Primary open angle glaucoma patients and glaucoma suspects, showed significant reduction in depth perception. Decreased stereoacuity was associated with greater glaucomatous visual field loss.

## Introduction

Glaucoma is a progressive optic neuropathy which leads to irreversible functional loss of vision. Currently, perimetry is the standardized tool for assessing glaucoma progression since visual field loss is the most common form of functional vision loss associated with glaucoma [1].

Depth perception- ability to see things in three dimensions-is one of the higher grades of binocular function that requires cortical integration [2–4]; in performing day to day activities such as grasping, distance judgment and driving [5, 6]. Retinal ganglion cells (RGC) of magnocellular pathway that correlates with stereoscopic pathway are involved in glaucoma [5]. The progressive loss of RGC in patients with glaucoma can be correlated with decreased depth perception seen in glaucoma. This structural basis of stereoacuity deficits was explored in one study and found that with decrease in RNFL thickness, stereoacuity both for distance and near decreases [7]. Studies have shown that decrease in stereopsis effects the quality of life in them. There have been very few studies, which have evaluated stereoacuity as a part of functional impairment in glaucoma. Depth perception deficit may precede visual field loss in glaucoma [5]. The studies done in past in glaucoma patients reporting depth deficits means ranged from 118 to 494 arc sec [3, 7–10]. More so, only few studies have examined stereopsis in glaucoma and suspects as well, and two of them have reported stereopsis deficits [2, 3, 9] However, in one study stereopsis deficits were not detected in glaucoma suspects [2]. The tools for stereoacuity measurement have also been different in them.

In routine clinical practice beside visual fields, optical coherence tomography of optic nerve head is done to diagnose and monitor progression in glaucoma but binocular function tests like stereopsis is not done regularly. Since glaucoma is a progressive disease, assessing the stereo-acuity might add additional information of functional loss of vision early in the course of disease. The unveiling of preperimteric glaucoma with this functional test is still debatable but testing stereopsis might be useful to look at the qualitative aspect of vision which is necessary in day-to-day activities. Screening of stereopsis with both global and local real depth stereopsis in same patient might reveal threshold variability among test tools too. Therefore in this study ,we aimed to find association of impaired depth perception measured with both Titmus and Frisby stereoacuity test in glaucoma patients and suspects to determine how this test is able to evaluate the glaucomatous damage compared to age and sex matched controls. We have also tied to investigate the association of severity of POAG and stereoacuity.

### Methodology

#### Study area and period

The study was done at BP Koirala Lions Centre for Ophthalmic Studies (BPKLCOS), Maharajgunj, Nepal. BPKLCOS is a tertiary eye care center established under the umbrella of Institute of Medicine -one of the premier medical institutes of Nepal. It is also referral center where teaching learning activities of medical and optometry and visual science students of Trivhuvan University takes place.

#### Study design

This was a hospital based comparative cross-sectional study. Ethical clearance was taken from Institute of Medicine- Institutional Review committee [Reference no.399(6-11)E^2^ 077-078]. The study was carried out for six-month period from January 2021-June 2021.

#### Eligibity Criteria

After taking informed as well as written consent, all patients meeting inclusion criteria (patient of ≥ 40 years,orthotropic by near and far on cover uncover tests with a minimum best corrected visual acuity of 6/12 in each eye taken with Snellen, differing by no greater than one Snellen acuity line, and no history of incisional surgery within the previous 3 months) were be subjected to stereopsis assessment with Titmus and Frisby test. While patients of neurological disease, corneal opacity that might interfere with test, visually significant cataract (As per lens opacity classification system (LOCS II, >NSII, PSCC>II, Cortical>II), strabismus or strabismus surgery, amblyopia, diabetic retinopathy were excluded from study.

#### Sample size and Sampling procedure.

Sample size size of 20 for each h group was calculated using the standard formula for sample size in a comparative study with 95% confidence interval and error with 10% tolerance where prevalence of glaucoma was taken from study conducted in Nepal [11]. Purposive sampling technique was utilized to select t the study participants.

#### Data collection procedure

Detailed history and clinical examination were done with including visual acuity, anterior and posterior segment evaluation along with cover uncover test. Each group consisted of 20 participants meeting inclusion and exclusion criteria. Patients included in the study were examined by glaucoma specialists in Glaucoma clinic at BPKLCOS. Data was filled in preformed proforma. Information on age, gender, intraocular pressure, cup-disc ratio, central corneal thickness, mean visual field score and stereopsis measured with Titmus and Frisby stereopsis test were recorded.

#### Data processing and analysis

The collected data was cleaned, entered, and analyzed using Statistical analyses were performed using SPSS (Statistical Package for Social Sciences; SPSS Inc. IBM) version 21.0. Data are expressed as the means ± standard deviation for age, IOP, CCT, stereopsis. The Spearman correlation was done between age and stereoacuity. The difference in stereoacuity in control and other groups were done with paired t-test. The difference in stereoacuity between titmus and frisby stereoacuity tests and VFI score was measured by Anova Test.

#### Visual acuity measurement

Vision was taken with Snellen distance vision drum.

#### Glaucoma Definition

In this study primary open angle glaucoma was defined by characteristic glaucomatous optic nerve head findings a with the presence of corresponding visual field deficits in one or both eyes with open anterior chamber angles on gonioscopy. The diagnosis of glaucoma was established by Hagstreit USA slitlamp biomicroscope (BQ-900), Zeiss 4 mirror gonioscopy, intraocular pressure measurement by Goldman applanation tonometer and fundus examination using the Volk 90D indirect lens to evaluate the optic disc. All patients’ visual fields were assessed by the static perimeter Humphrey visual field analyzer II; using central 24-2 program, Swedish interactive threshold algorithm (SITA) Fast protocol with stimulus size III, white object, (24-2 central). Glaucoma suspects were required to have a normal Humphrey Sita Fast 24-2 visual field with optic nerve head findings suspicious for glaucoma. All control subjects were age matched, with no incisional surgical history, and underwent a full eye examination. Control subjects were recruited from hospital personnel or accompanying relatives of patients, and had normal eye examinations.

#### Disease Severity

We documented both visual field index (VFI) and mean deviation (MD) in all of our patients.

VFI, which ranges from ranging from 0 to 100% signifies vision ranging from blindness to normal respectively, expresses visual function as a percentage of a perimetrically normal age-corrected visual field and is used for calculation of rate of progression by regressing VFI over time. The worse eye and other eye on the VFI were divided into three glaucomatous groups as mild (>67 percentile, 68–95%), moderate (34–66 percentile, 35–67%) or severe defect (<33 percentile). Near stereoacuity is influenced by parafoveal visual defects. In VFI the paracentral points are given more importance than are the peripheral points and which is generally affected in glaucoma so this grading was done for severity assessment. More so, glaucoma has asymmetric presentation in eyes so if there was a difference in the severity of VF defects between participants’ eyes, the worse eye was used VFI grading

### Titmus test

The Titmus stereo test consists of a combination of contour targets. The most common targets are a series of rings for older patients, animals for children, and a large stereo fly that is used mainly for screening. The fly measures a gross stereoacuity of 3000 arc sec while the test circles measure finer stereoacuity ranging from 800 to 40 arc sec. Subjects have to wear polarized lenses over their existing spectacle correction (if any) and will be initially screened using the fly followed by stereo test circles at the standard testing distance of 40 cm under normal lighting condition. The normal near stereoacuity is approximately 20 (+10) arc sec. Subjects with stereoacuity of 40 arc sec will be graded as normal, >40 arc sec were graded as reduced and failure to perceive the depth in the fly test (>3000 arc sec) were considered to have absence of gross stereoacuity.

### Frisby stereo test

Stereoscopic visual acuity will be assessed using the Frisby stereo test (Clement Clarke International Ltd, Essex, UK) according to the instructions of the manufacturer. The Frisby stereo test consists of three plates of Perspex glass measuring 1 mm, 3 mm, and 6 mm in thickness. This test was performed for all patients under the same conditions. The subject response and stereoacuities will be recorded with a range of values from 20 seconds of arc to 340 seconds of arc. The lowest disparity which the patient can reliably discriminate will be recorded and this stereo-threshold is a measure of stereoacuity. The 6 mm plate will be shown at a viewing distance of 80 cm. If the answer was correct, the 3 mm plate was presented at the same distance. If the answer was incorrect for the 3 mm plate, a score of 85 seconds of arc was recorded. If the answer was correct, the 1 mm plate was presented. If the answer was incorrect, a score of 40 seconds of arc was recorded, and if the answer is correct, a score of 20 seconds of arc will be recorded. If the answer was incorrect for the 6 mm plate at 80 cm, the same plate was viewed at a distance of 60 cm. If the answer was correct, a score of 150 seconds of arc was recorded; while if the answer is incorrect, the 6 mm plate will be presented at 40 cm. If the answer was correct, a score of 340 seconds of arc was recorded. If the subject could not detect the quadrant with depth effect at this distance, the highest stereo-threshold value tested (340 seconds of arc) was assigned

## Result

### Demographic Characteristic

There were no differences in age, sex, or best corrected visual acuity, intraocular pressure, central corneal thickness (CCT), found among the three groups (POAG, Glaucoma Suspects and Control) respectively. However, there was significant difference in cup disc ratio (CDR) between the groups. Equal number of male and female were there in each group, while in POAG group male to female ratio was 3:2 (Table 1).

**Table 1:** Demographic characteristics of control, suspect, and POAG groups

	Control	Glaucoma Suspect	POAG	Significance
**Mean Age**	**51.1±3.16**	**46.55±2.58**	**54.85±3.67**	**ANOVA,** ***p*** **= 0.189**
**Sex(F:M)**	**1:1**	**1:1**	**2:3**	**chi-square,** ***p*** **= 0.765**
**Smoking**	**5**	**3**	**2**	**ANOVA,** ***p*=0.065**
**Diabetes Mellitus**	**2**	**3**	**3**	**ANOVA,** ***p*=0.872**
**Hypertension**	**5**	**6**	**6**	**ANOVA,** ***p*=0.092**
**Family history of glaucoma**	**1**	**5**	**3**	**ANOVA,** ***p*=0.216**
**Mean Best corrected Vision**	**6/6**	**6/6-6/9**	**6/6-6/9**	**chi-square,** ***p*** **= 0.529**
**Mean IOP**	**14.5±0.5**	**15.5±0.93**	**13.63±0.79**	**ANOVA,** ***p*** **= 0.314),**
**Mean CCT**	**535±5**	**542.87±15.5**	**536.12±9.3**	**ANOVA,** ***p*** **= 0.915**
**Mean CDR**	**0.25±0.14**	**0.62±0.02**	**0.75±0.02**	**ANOVA,** ***p*=0.001**

### Stereoacuity among study participants

The mean stereoacuity threshold among control group, glaucoma suspects and POAG are shown Table 2. There was significant difference in threshold between groups with Titmus (ANOVA, *p* = 0.019 and by Frisby (ANOVA, *p* = 0.023) Tests.. There was significant difference in stereoacuity measured by Titmus to that of Frisby in between groups (*p*=,0.05) by ANOVA one way test. The difference in threshold was significant between control and glaucoma suspects with Titmus (t=1.991, *p*=0.05) and with Frisby (t=2.114, *p*=0.04). The difference was also significant between control and POAG group by Titmus (t=3.135, *p*=0.0033) and by Frisby (t=3.014, *p*=0.004), at 95%CI Table 2.

**Table 2:** Stereoacuity threshold characteristics between Groups

Diagnosis		STREOTITMUS	STEREOFRISBY
**Control**	**Mean (sec of arc)**	**53.50**	**38.75**
	**Std. Deviation**	**23.23**	**18.83**
	**Minimum**	**30.00**	**20.00**
	**Maximum**	**100.00**	**85.00**
**GS**	**Mean (sec of arc)**	**175.00**	**135.00**
	**Std. Deviation**	**271.96**	**202.70**
	**Minimum**	**40.00**	**20.00**
	**Maximum**	**800.00**	**600.00**
**POAG**	**Mean (sec of arc)**	**277.00**	**199.75**
	**Std. Deviation**	**317.95**	**238.12**
	**Minimum**	**40.00**	**40.00**
	**Maximum**	**800.00**	**600.00**

### Correlation of stereoacuity thresholds with age and in between tests

The Spearman correlation between age and stereoacuity was 0.463, 0.484 (*p* < 0.01) with Titmus and Frisby respectively. Titmus test , showed positive correlation Pearson correlation, *p* < 0.001.with Frisby test.

### Mean defect and Visual field Index

The mean VFI score among right eyes (*n*=60) was 91.47 % and left eyes(*n*=60) was 90.03% while mean Mean defect was -3.75db in right eyes and -3.78 in left eyes as shown in Table 3.

**Table 3:** Mean defect and Visual field Index in right eye and left eye

	Minimum	Maximum	Mean	Std. Deviation
**VFIRE(%)**	**10.00**	**100.00**	**91.4767**	**18.95415**
**VFILE(%)**	**18.00**	**100.00**	**90.0333**	**18.25615**
**MDRE (db)**	**-33.53**	**1.15**	**-3.7580**	**6.71628**
**MDLE (db)**	**-25.33**	**1.80**	**-3.7827**	**5.96552**

The inter eye difference in severity of visual field index (better and worse eye) is shown in control,glaucoma suspects and POAG group are shown in Table 4.

**Table 4:** The inter eye difference in severity of visual field index (better and worse eye)

Group		Better Eye		Worse Eye	
	**VFI severity**	**Frequency**	**Percentage**	**Frequency**	**Percentage**
**Control**	**Normal**	**20**	**100**	**20**	**100**
**Glaucoma Suspects**	**Mild**	**3**	**15**	**6**	**30**
	**Normal**	**17**	**85**	**14**	**70**
**POAG**	**Mild**	**5**	**25**	**7**	**35**
	**Moderate**	**3**	**15**	**6**	**30**
	**Normal**	**10**	**50**	**4**	**20**
	**Severe**	**2**	**10**	**3**	**15**

All of the control had normal VFI score, while 70% of the glaucoma suspects had normal VFI score whereas only 20% of the POAG group had normal VFI.

### Association between VFI score worse eye and stereopsis thresheshold

Among the study patients 38 patients had normal VFI score while 13 had mild, 6 had moderate and 3 had severe VFI score on worse eye. Worse eye VFI score was 100% in normal range in control group,70% normal and 30% mild glaucomatous range in glaucoma suspect. While in POAG group, it . was 20% in normal ,35% in mild glaucomatous ,30% in moderate and 15% in severe glaucomatous range .The distribution of worse eye VFI score along with steroaquity threshold is shown in Table 5. With increasing severity of glaucoma, with a decrease in VFI score; the mean threshold of stereopsis increased as seen with both Titmus and Frisby Tests (ANOVA, *p* < 0.001).

**Table 5:** Distribution of VFI score on worse eye and mean steroauity threshold among the groups

Diagnosis	VFI		STEREOTITMUS	STEREOFRISBY
**Control**	**Normal**	**N**	**20**	**20**
		**Mean**	**53.5000**	**38.7500**
		**Std. Deviation**	**23.23224**	**18.83970**
**GS**	**Mild**	**N**	**6**	**6**
		**Mean**	**310.0000**	**241.6667**
		**Std.eviation**	**380.26307**	**278.29241**
	**Normal**	**N**	**14**	**14**
		**Mean**	**117.1429**	**89.2857**
		**Std. Deviation**	**201.16693**	**150.88020**
**POAG**	**Mild**	**N**	**7**	**7**
		**Mean**	**75.7143**	**62.8571**
		**Std. Deviation**	**25.72751**	**21.76717**
	**Moderate**	**N**	**6**	**6**
		**Mean**	**508.3333**	**345.8333**
		**Std. Deviation**	**332.29003**	**279.14900**
	**Normal**	**N**	**4**	**4**
		**Mean**	**75.0000**	**60.0000**
		**Std. Deviation**	**30.00000**	**23.45208**
	**Severe**	**N**	**3**	**3**
		**Mean**	**553.3333**	**413.3333**
		**Std. Deviation**	**427.23920**	**323.31615**

## Discussion

This study showed that stereoacuity was reduced among glaucoma and suspects compared to control and this increase in the threshold correlated with disease severity.

In our study, we had taken stereopsis with two different tests used widely in all the groups and tried to correlate the results. Frisby test of stereopsis presents real depth to the subject in the form of different plate thicknesses, while the Titmus test uses black, contoured stimuli, with Polaroid glasses to separate the stimuli presented to each eye. Variation in stereoacuity thresholds has been observed in individual subjects between stereo tests in past [12], but recently it has been found that the stereoacuity is stable regardless of the test graphs used (contour-based or random-dot based) or the test distance (far or near) in a population with normal stereopsis [13]. Comparable findings were seen in between the test with slight overestimation of threshold with Frisby compared to Titmus.

Few studies [14, 15] have reported that the stereoacuity worsened with increasing age. In another study, no statistically significant difference was found with age and in regard to stereopsis threshold [16]. In our study, there was significant correlation with age and stereoacuity threshold. Beside age, lens changes [17] also has an effect on stereoacuity, here in our study however we did not took the status of lens into account as cases with significant cataract were excluded from the study and lenticular opacity grading was not correlated with stereopsis.

In our study the mean stereoacuity threshold (seconds of arc) in control group was 53.5±23.23 with Titmus and 38.75±18.83 with Frisby stereoacuity tests while in glaucoma suspects it was 175+-271 with Titmus and 135+_202.70 with Frisby stereoacuity tests . Similarly, in POAG it was 277+_317 with Titmus and 199.75+_238.12 with Frisby stereoacuity tests. The difference in threshold was significant between control and glaucoma suspect as well as POAG group with both the steoacuity tests. In par with our study ,significant reduction in both near and distance stereoacuity was observed in the glaucoma group compared with the control group in study done by byYoshikawa T et al [18]. The mean stereo threshold in glaucoma patients was also increased compared to age matched normal (148.1 v 26.6 seconds of arc; *p* = 0.0004 )in another study [3]. Similarly, stereopsis for the normal tension glaucoma patients were decreased compared to the normal controls in another study where stereopsis was performed using Titmus stereo test [19]. Patients in mild glaucoma group also showed statically significant depth perception defects as compared to the controls in another study [5]. Findings of these studies may suggest the disrupted binocular vision early on the disease course.

 laucoma suspects showed significantly increased mean stereo threshold compared to age matched normal [3] which was also seen with glaucoma suspects in our study too. The increased threshold of stereopsis in glaucoma suspects may also suggest pre-perimetric visual dysfunction in these group. So decreased stereopsis may be a precursor of overt glaucomatous damage.

In our study, with decrease in VFI score or with severity of POAG, mean threshold increased, indicating with increasing severity of glaucoma stereoacuity decreases though the number of severe diseases were less. In a similar study, decreased median stereoacuity was associated with greater glaucomatous visual field loss where subjects were classified with both Hodapp-Anderson-Parrish (HAP) and VFI as mild, moderate and severe [10]. The depth perception defects were also increased in advanced glaucoma group with a statically significant difference between mild and advanced groups, in a cross- sectional study who underwent Lang stereoacuity test done by Elgohary et al [5]. In another cross-sectional study done by Pradeep N, et al [20] where stereopsis was done using stereo fly test, stereopsis was seen to be grossly affected with glaucoma progression. Similarly, in another study done to assess the associations between stereoacuity (both near and distance) and the severity of visual field defects (based on HAP and VFI) in POAG, near stereoacuity worsened with POAG severity. However, distance stereoacuity was not associated with POAG severity [21]. From these, it could be interpreted that impaired depth perception seen in POAG patients is related to the disease process, which could lead to difficulties in activities of daily living. So, more information can be gathered with stereopsis test while assessing patients along with other binocular dysfunction in glaucoma cases as severity increases.

### Limitations

Being a cross-sectional study, we could not evaluate whether reduced steroaquity threshold increases with disease progression on follow up in same patients. A longitudinal study in this aspect could be explored since glaucoma is a progressive disease. More so in our study we only analyzed the VFI of the worse eye and the binocular visual fields were not taken into account so comparison with severity couldnot be fully established as stereopsis is a binoocular phenomenon and binocular difference in the severity of glaucoma may affect the binocular interaction and binocular function.

Effect of threat to fovea or fixation, field defects in superior or inferior hemifield were not studied too which might have effect on stereopsis.

## Conclusion

We found that Primary open angle glaucoma patients and glaucoma suspects, showed significant reduction in depth perception measured with both Titmus and Frisby Depth Test, which may have utility in identifying early glaucomatous nerve damage. Test of stereopsis along with other binocular visual function test may help in uncovering early disease . With increasing severity of Glaucoma, depth perception decreases, this reduction may help in assessing difficulties faced in daily activities in advance glaucoma cases.

In clinical practice,assessing stereopsis and other binocular vision tests might add additional information for glaucoma care and may serve as preperimetric functional tool. It should be routionely done in glaucoma patients and suspects.

## Data Availability

The data that support the findings of this study are available on request from the corresponding author

## Abbreviations

POAG: Primary open angle glaucoma
CDR: Cup disc ratio
VFI: Visual field index
HAP: Hodapp-Anderson-Parrish
RNFL: Retinal nerve fiber layer
BPKLCOS: BP Koirala Lions Centre for Ophthalmic Studies
